# Automated measurement of field crop phenotypic traits using UAV 3D point clouds and an improved PointNet++

**DOI:** 10.3389/fpls.2025.1654232

**Published:** 2025-09-12

**Authors:** Jiatong Yao, Wei Wang, Hongyu Fu, Zhehong Deng, Guoxian Cui, Shuaibin Wang, Dong Wang, Wei She, Xiaolan Cao

**Affiliations:** ^1^ College of Information and Intelligence, Hunan Agricultural University, Changsha, China; ^2^ College of Agriculture, Hunan Agricultural University, Changsha, China; ^3^ Hunan Cultivated Land and Agricultural Eco-Environment Institute, Changsha, China; ^4^ Technology Center, China Tobacco Hunan Industrial Co., Ltd, Changsha, China

**Keywords:** UAV remote sensing, 3D point cloud, deep learning, phenotypic trait extraction, stem-leaf segmentation

## Abstract

Accurate acquisition of tobacco phenotypic traits is crucial for growth monitoring, cultivar selection, and other scientific management practices. Traditional manual measurements are time-consuming and labor-intensive, making them unsuitable for large-scale, high-throughput field phenotyping. The integration of 3D reconstruction and stem–leaf segmentation techniques offers an effective approach for crop phenotypic data acquisition. In this study, we propose a tobacco phenotyping method that combines unmanned aerial vehicle (UAV) remote sensing with an improved PointNet++ model. First, a 3D point-cloud dataset of field-grown tobacco plants was generated using multi-view UAV imagery. Next, the PointNet++ architecture was enhanced by incorporating a Local Spatial Encoding (LSE) module and a Density-Aware Pooling (DAP) module to improve the accuracy of stem and leaf segmentation. Finally, based on the segmentation results, an automated pipeline was developed to compute key phenotypic traits, including plant height, leaf length, leaf width, leaf number, and internode length. Experimental results demonstrated that the improved PointNet++ model achieved an overall accuracy (OA) of 95.25% and a mean intersection over union (mIoU) of 93.97% for tobacco plant segmentation—improvements of 5.12% and 5.55%, respectively, over the original PointNet++ model. Moreover, using the segmentation results from the improved PointNet++ model, the predicted phenotypic values exhibited strong agreement with ground-truth measurements, with coefficients of determination (R²) ranging from 0.86 to 0.95 and root mean square errors (RMSE) between 0.31 and 2.27 cm. This study provides a technical foundation for high-throughput phenotyping of tobacco and presents a transferable framework for phenotypic analysis in other crops.

## 1 Introduction

Tobacco, as one of the world’s major economic crops, requires accurate plant phenotypic parameters for cultivar improvement and optimization (Koh et al., 2021). Traditional tobacco phenotyping data collection primarily relies on manual measurements, which are time-consuming, costly, prone to human error, and may damage plant morphology, making them unsuitable for large-scale phenotypic analysis tasks (Kaiser et al., 2018). UAV remote sensing technology addresses the limitations of traditional phenotyping methods by offering advantages such as rapid, non-destructive, and flexible data collection, becoming a promising tool for acquiring field crop phenotypic information. Using multi-view remote sensing imagery captured by UAVs, combined with 3D reconstruction technology, it is possible to obtain the complete geometric structure of plants in a non-contact manner, enabling refined phenotypic parameter extraction through plant segmentation.

In UAV-based plant phenotyping studies, stem-leaf segmentation remains a challenging task (Chen et al., 2025). Traditional segmentation methods primarily rely on techniques such as region growing, attribute clustering, and skeleton extraction. For instance, Li et al. proposed a leaf segmentation method for dense plant point clouds based on small planar region growing, which involves three steps: point cloud preprocessing, over-segmentation of planar regions, and region growing, to achieve individual leaf segmentation in greenhouse ornamental plants (Li et al., 2018). Ferrara et al. employed the DBSCAN density-based clustering algorithm to analyze plant point clouds collected by LiDAR, enabling the automatic separation of tree leaves and trunks (Ferrara et al., 2018). Sun et al. constructed a dual-threshold segmentation approach using the Otsu algorithm, based on plant height and reflectance intensity in different parts of rice panicles, and optimized point cloud processing through super pixel and mean-shift clustering methods (Sun et al., 2021). Although these methods have achieved certain success in plant stem-leaf segmentation, they heavily depend on manually defined rules, involve high computational complexity, and are difficult to scale for high-throughput phenotyping. With the advancement of deep learning, its end-to-end feature extraction and adaptive optimization capabilities offer a more robust and computationally efficient solution for plant stem-leaf segmentation.

Deep learning-based segmentation methods are generally categorized into four types: projection-based, voxel-based, graph-based, and point-based approaches (Sarker et al., 2024). Among these, projection, voxelization, and graph-based methods require the transformation of point cloud data into regular grid or graph structures (Shi et al., 2019; Yang et al., 2021; Saeed et al., 2023). However, these transformations often lead to the loss of three-dimensional information and fine details during processing (Das Choudhury et al., 2020; Zięba-Kulawik et al., 2021). And may also introduce instability in adjacency construction and feature propagation (Phan et al., 2018; Mirande et al., 2022).

In contrast, point-based methods operate directly on raw point cloud data without requiring spatial transformations, allowing for better preservation of original geometric information. This advantage is particularly important in point cloud segmentation tasks, where maintaining fine-grained local geometric features is essential. Point-based methods also effectively avoid the detail loss issues commonly associated with voxelization and graph construction. In addition, these methods typically require fewer computational resources and are well-suited for handling complex and irregular point cloud structures, making them increasingly popular in plant phenotyping applications (Turgut et al., 2022; Yang et al., 2024).

In the field of tobacco phenotypic trait extraction, the application of these methods remains relatively limited. Existing approaches primarily rely on depth cameras or LiDAR to obtain point cloud data of individual tobacco plants, followed by segmentation using clustering algorithms. However, such methods often struggle with insufficient feature extraction capability, low computational efficiency, and uneven point cloud density when processing the detailed structures of tobacco organs (Briechle et al., 2020; Zhu et al., 2023). Furthermore, variations in plant growth stages introduce additional noise and structural changes in the point cloud data, further affecting segmentation accuracy. To address these challenges, this study proposes a deep learning-based method for tobacco organ segmentation and phenotypic trait computation. The main contributions are as follows:

1. In this study, we constructed a tobacco point cloud dataset to enhance model robustness. A 3D point cloud model of tobacco plants was generated using multi-view UAV image acquisition, followed by point cloud preprocessing and manual annotation. This dataset improves the uniformity of point cloud density and contributes to more accurate phenotypic analysis.
2. We proposed an improved PointNet++ model. Building upon the original PointNet++ architecture, the model adopts a multi-layer feature extraction strategy and integrates a Local Spatial Encoding (LSE) module and a Density-Aware Pooling (DAP) module. First, a multilayer perceptron (MLP) is used to learn basic point cloud features. Then, the LSE module captures local spatial relationships to enhance the representation of complex plant structures. Finally, the DAP module adaptively selects pooling strategies based on neighborhood point cloud density, improving the fusion of salient local features and addressing the limitations of traditional methods in feature extraction.
3. We integrated stem-leaf segmentation with phenotypic parameter computation to improve the efficiency of phenotypic data acquisition. This method extracts phenotypic parameters, including plant height, leaf length, leaf width, leaf number, and internode length. In the field of tobacco, a phenotypic parameter calculation method has been proposed, which successfully resolves the contradiction between measurement accuracy and efficiency in phenotypic parameter determination.

## 2 Materials and methods

**Figure 1:**
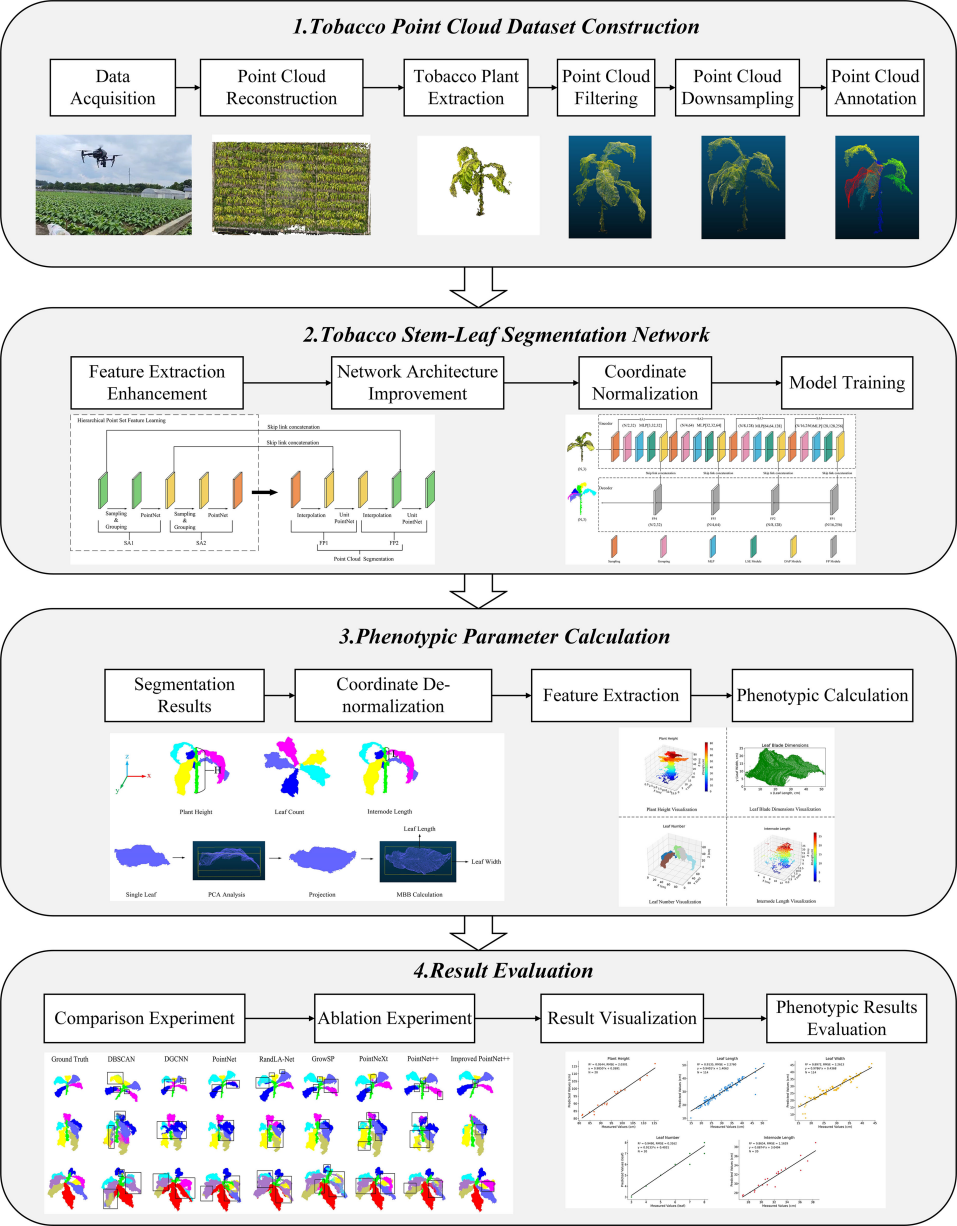
Overall workflow for calculating tobacco phenotypic parameters.

This study is divided into the following four main parts (**Figure 1**):

1. Tobacco point cloud dataset construction: Multi-view images of tobacco plants were collected using a UAV, and a 3D point cloud model was reconstructed. Individual plant extraction and point cloud preprocessing were performed to provide a data foundation for model training.
2. Tobacco stem-leaf segmentation network: An improved network based on PointNet++ was proposed. Coordinate normalization was first applied to the input point cloud to standardize spatial information, followed by enhancement of the feature extraction module to improve the segmentation performance of the model for tobacco stem and leaf structures. The model was then trained accordingly.
3. Phenotypic parameter calculation: Based on the segmentation results, coordinate de-normalization was performed to restore the actual scale, and phenotypic traits such as plant height, leaf length, leaf width, leaf number, and internode length were calculated.
4. Result evaluation: Evaluation was conducted from two perspectives: segmentation performance and phenotypic accuracy. The segmentation results were validated through comparisons with mainstream models and ablation experiments, while the accuracy of phenotypic parameter estimation was verified against measured data.

### 2.1 Dataset and collection

#### 2.1.1 Experimental area

**Figure 2:**
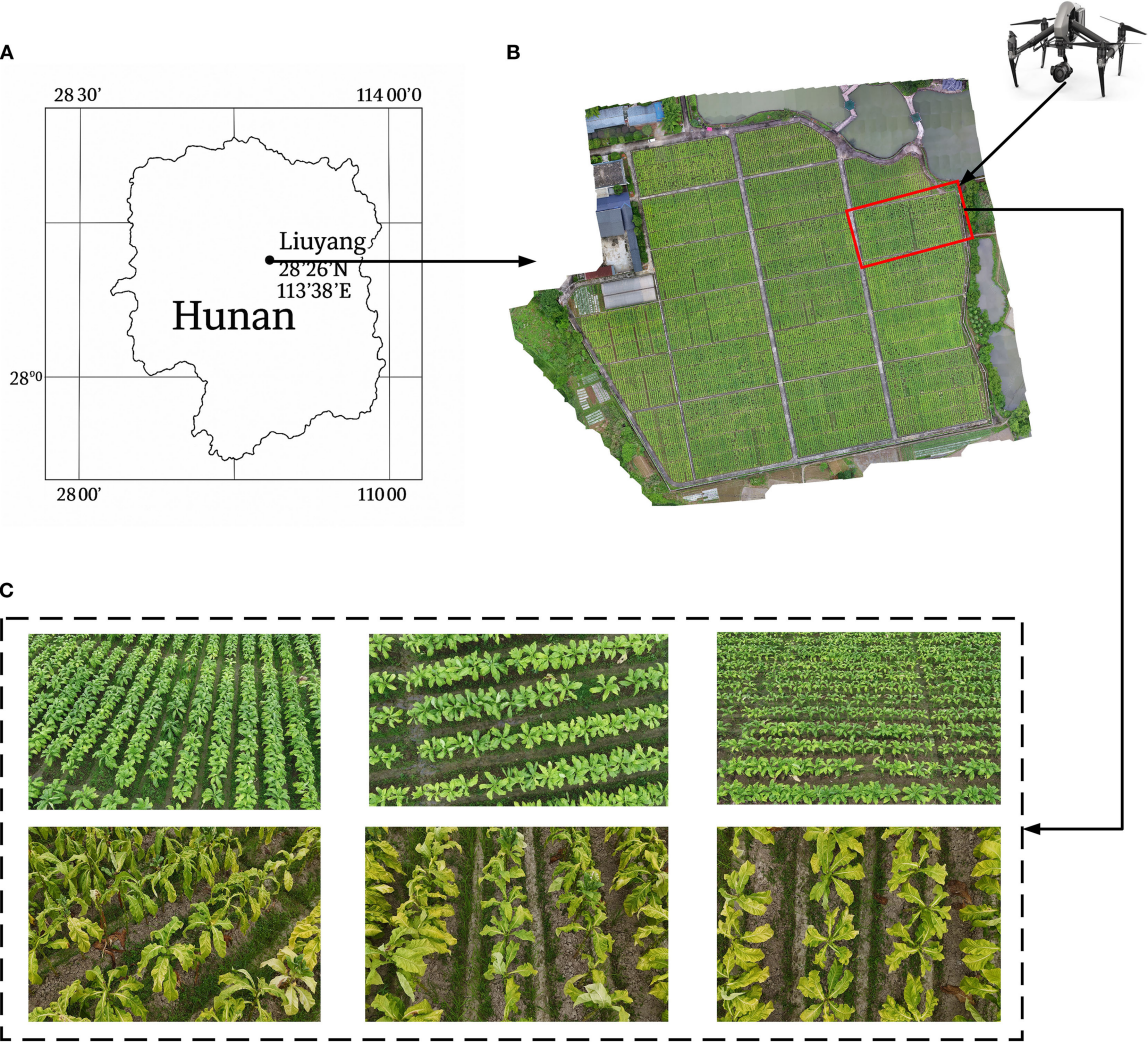
Tobacco image data collection. **(A)** schematic of the experimental base; **(B)** experimental data collection area; **(C)** UAV captured multi-view tobacco images during two different periods.

The experimental area is located at the Guandu Experimental Base of the Hunan Tobacco Technology Center in Liuyang, Hunan Province (**Figure 2**). This region has a subtropical monsoon humid climate with abundant rainfall and favorable light and heat conditions. A total of 400 tobacco breeding materials were planted in the experimental area. Tobacco seedlings were transplanted in March 2024 with a row spacing of 120 cm and a plant spacing of 50 cm.

#### 2.1.2 Data collection

Data collection for this study was conducted at two critical developmental stages of tobacco: the peak growth stage (June 23, 2024) and the post-topping stage (July 13, 2024) (**Figure 2**). The data types included UAV imagery and detailed ground-based phenotypic measurements, all collected on the same day for each stage.

(1) UAV imagery data

The image data were acquired using a DJI Inspire 2 unmanned aerial vehicle (UAV) equipped with a Zenmuse X5s camera (35 mm focal length). The camera has an effective pixel count of 20.8 million and a maximum resolution of 5280×3956. The UAV flew at a height of 5 meters and captured images of the tobacco plants at 30°, 60°, and 90° angles. The flight speed was set to 2 meters per second, resulting in a total of 2220 images collected (**Figure 2**).

(2) Ground-based tobacco phenotypic data

Phenotypic data were obtained through manual measurements, including the true values of tobacco plant height, leaf length, leaf width, leaf number, and internode length. During measurement, plant height was defined as the straight-line distance from the ground to the highest leaf; leaf length was the straight-line distance from the point where the leaf connects to the stem to the leaf tip; leaf width was measured as the maximum width perpendicular to the main vein direction; leaf number was determined by manual counting; and internode length was defined as the vertical distance between the base of the upper and middle leaves.

### 2.2 Tobacco point cloud model reconstruction and processing

#### 2.2.1 Tobacco point cloud model reconstruction

This study employs a multi-view image-based reconstruction technique, using Structure From Motion (SFM) and Multi-View Stereo (MVS) methods to construct the tobacco point cloud model (Luo et al., 2024). The reconstruction process includes the generation of sparse point clouds and the reconstruction of dense point clouds (Gonçalves et al., 2021). The entire reconstruction process was done using the structure-from-light measurement software Agisoft Metashape (version 2.1.2).

**Figure 3:**
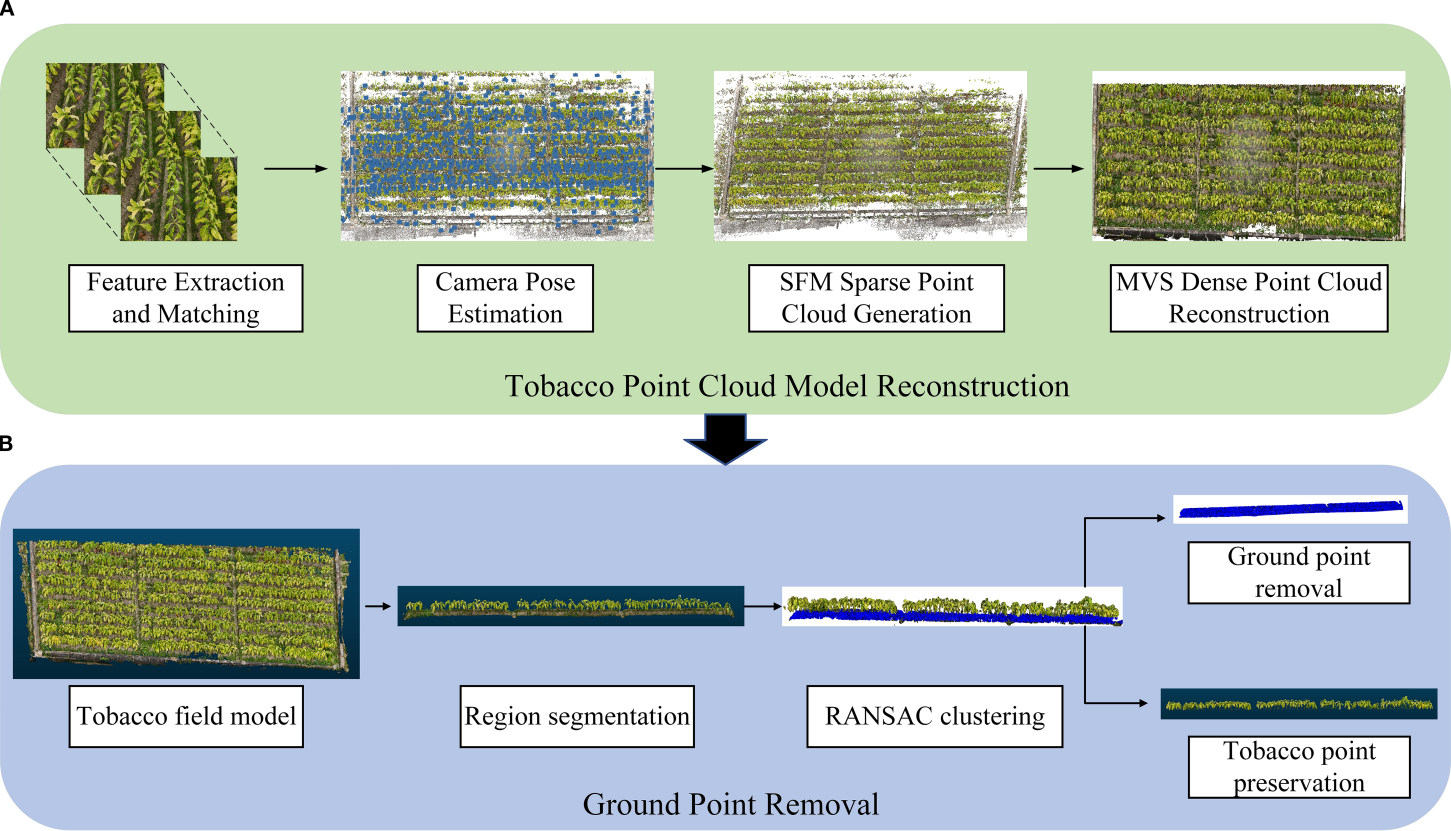
Overall workflow of tobacco model reconstruction and preprocessing. **(A)** Tobacco Point Cloud Model Reconstruction; **(B)** Ground Point Removal.

The SFM-MVS workflow includes the following steps (Gao et al., 2022) (**Figure 3**): (1) Feature Extraction and Matching: Key feature points are extracted from the input multi-view tobacco images, and feature matching relationships are established between the images. (2) Camera Pose Estimation: Based on the feature matching results, the camera’s position and orientation are estimated for each capture location. (3) Sparse Point Cloud Generation: Using the camera poses and matched feature points, the 3D coordinates of the feature points are recovered through triangulation, generating the sparse point cloud. (4) Dense Point Cloud Reconstruction: The sparse point cloud is refined by increasing point density, fusing multi-view image information to generate a detailed dense point cloud.

#### 2.2.2 Ground point removal

Due to the large space occupied by ground points in the model and their frequent mixing with tobacco point clouds, it is necessary to remove the ground points. First, the entire tobacco model is segmented to reduce computational load (Ghahremani et al., 2021). Then, the Random Sample Consensus (RANSAC) algorithm is used to remove ground points (distance threshold: 0.2; number of points for fitting the plane: 3; number of iterations: 500) for ground point cloud removal (**Figure 3**).

The RANSAC algorithm iteratively selects random point sets to fit a plane model, and based on the distance from points to the plane, it identifies and segments ground and non-ground points (Harintaka and Wijaya, 2023). Specifically, three points are randomly selected to fit the plane model (Equation 1), the distance from each point to the plane is calculated (Equation 2), and ground points are identified based on a pre-set distance threshold. If the distance from a point to the plane is smaller than the threshold, it is classified as a ground point. Through multiple iterations, the plane with the most inliers is selected as the final ground model.


(1) ax+ by+cz+d=0

(2) $$d\left(p_{i}\right)=\frac{\left|ax_{i}+by_{i}+cz_{i}+d\right|}{\sqrt{a^{2}+b^{2}+c^{2}}}$$

where (a,b,c) represents the normal vector of the plane; (x,y,z) represents the coordinates of any point in the tobacco model; $p_{i}$ ​ represents the i -th point; and $d\left(p_{i}\right)$ represents the distance from the point to the plane.

#### 2.2.3 Point cloud denoising, downsampling, and labeling

**Figure 4:**
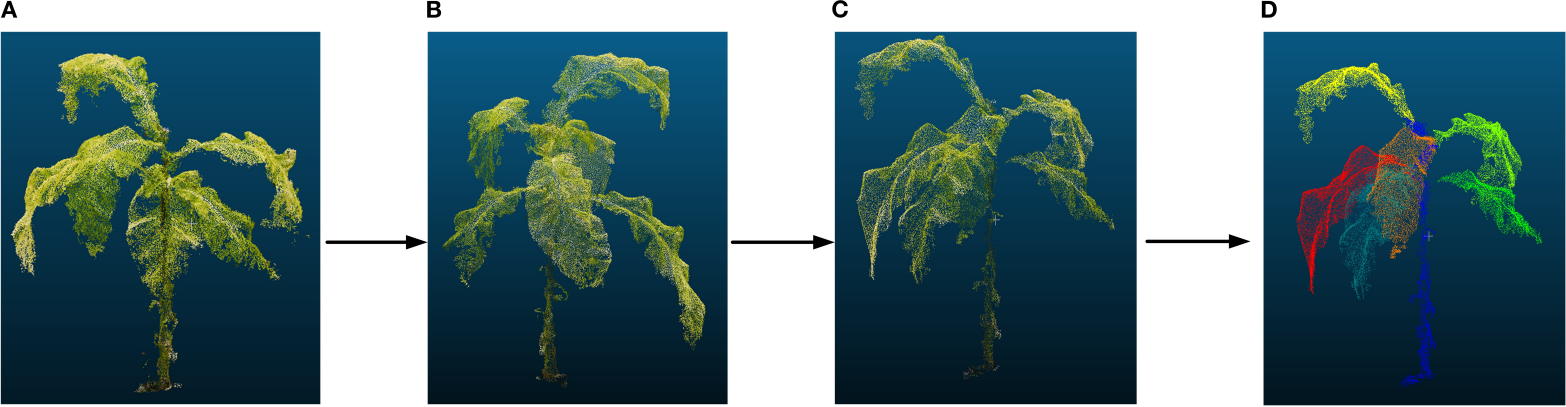
Individual tobacco plant extraction process. **(A)** individual tobacco plant; **(B)** statistical filtering; **(C)** uniform downsampling; **(D)** point cloud labeling.

After removing ground points, individual tobacco plants were extracted, and statistical filtering (neighboring points: 50; outlier threshold: 1.0) was applied for denoising to remove scattered points and improve data quality (Zhao et al., 2021). Uniform downsampling (sampling rate: 2) was performed to reduce the number of points and increase computational efficiency (Al-Rawabdeh et al., 2020). Additionally, based on the structural features of tobacco, point cloud data for the stem and leaves were manually labeled to clearly define the spatial position and geometric shape of the tobacco stem, which was used for model prediction comparison (**Figure 4**). Finally, region segmentation, individual plant extraction, and point cloud labeling were all conducted using CloudCompare software (version 2.14).

A total of 122 tobacco plants were labeled to construct the tobacco point cloud dataset. The training set included 102 plants, and the test set included 20 plants. **Table 1** presents the statistics for each category.

**Table 1:** Category statistics for training and test sets.

Category	Number of tobacco	Number of stems	Number of leaves	Number of stem points	Number of leaf points	Number of total points
Training Set	102	102	542	1479437	8901842	10381279
Test Set	20	20	114	295580	2002301	2297881

### 2.3 Tobacco stem-leaf segmentation network based on improved PointNet+

Although PointNet++ is capable of capturing local features through its hierarchical structure, its feature extraction accuracy remains limited when processing crop point clouds, especially for crops like tobacco with complex morphological structures. To enhance the segmentation performance of tobacco stems and leaves, this study introduces two key improvements to the original PointNet++: the integration of the LSE module and the incorporation of the DAP module.

The improved PointNet++ model adopts a multi-level feature extraction strategy. After extracting fundamental features using an MLP, the network enhances its feature representation at two hierarchical levels, as described below:

For enhancing local spatial information, traditional ball query grouping relies on a fixed radius, which may result in overly large or small local regions and thus fails to accurately capture fine-grained details of target structures in non-uniform point clouds. To address this limitation, this study adopts a K-Nearest Neighbors (KNN) grouping strategy and integrates the LSE module (Cheng et al., 2021). The LSE encodes local spatial relationships by computing the relative coordinates between each center point and its neighboring points. The encoded features are then concatenated with the original features and passed through an MLP to adjust the output dimension, thereby enhancing the representation of local spatial information (Hu et al., 2020; Chen et al., 2024).

For local feature aggregation, to enhance the model’s ability to extract features from non-uniform point clouds, this study incorporates the DAP module. It first computes the neighborhood density of each center point and then applies a relative density-based strategy to distinguish between high- and low-density regions. In high-density areas, max pooling is used to extract the most prominent local features, while in low-density regions, attention pooling is applied to perform weighted aggregation, emphasizing salient feature representation (Wu et al., 2022). Finally, the features from both areas are concatenated to obtain enhanced point cloud features, thereby improving the model’s segmentation performance in tobacco point cloud tasks (Deng et al., 2023).

#### 2.3.1 PointNet++ network architecture

**Figure 5:**
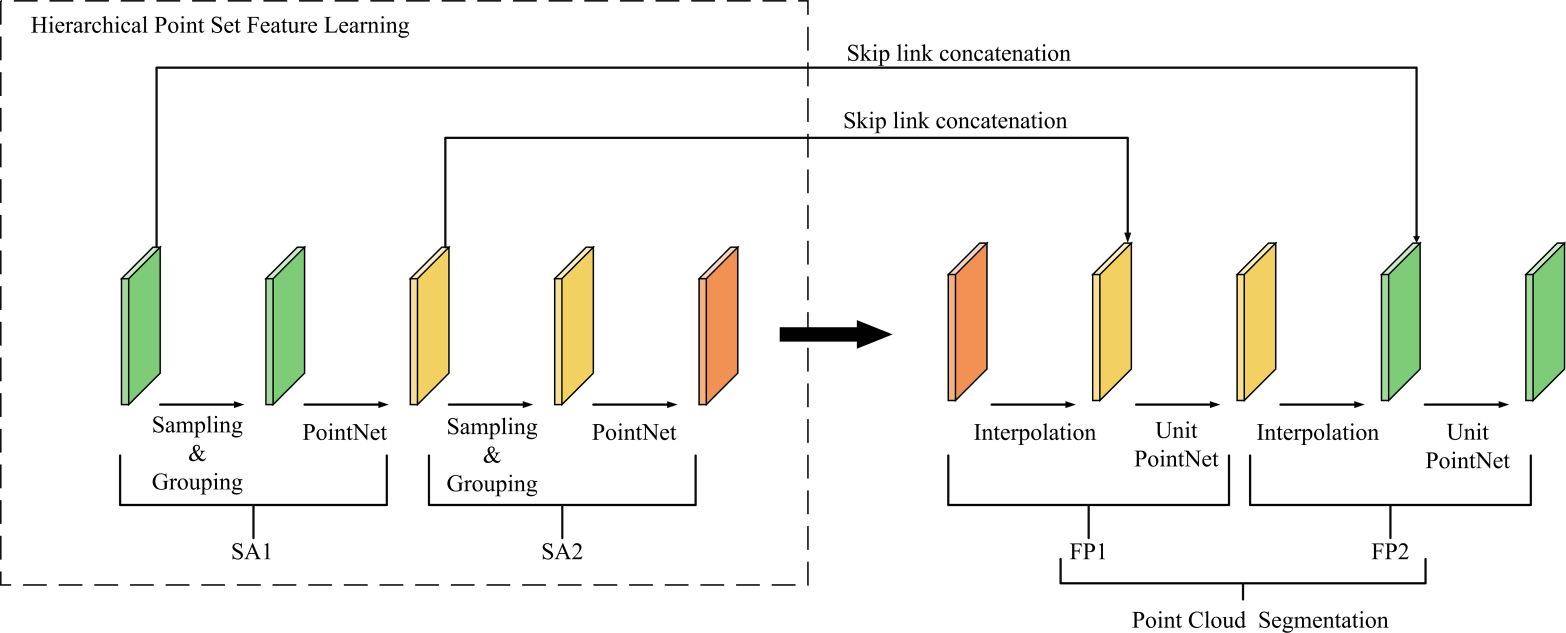
PointNet++ network architecture.

PointNet++ is a deep learning network designed for point cloud processing (**Figure 5**). As an improved version of PointNet, it enables hierarchical feature learning from point cloud data (Qi et al., 2017a).

The network architecture follows an encoder–decoder structure composed of multiple Set Abstraction (SA) modules and Feature Propagation (FP) modules. Each SA module consists of a sampling layer, a grouping layer, and a PointNet layer. The sampling layer adopts Farthest Point Sampling (FPS), the grouping layer constructs local regions using ball query, and the PointNet layer employs an MLP to extract features, followed by max pooling to aggregate global features. In the decoding stage, the FP module upsamples features through distance-weighted interpolation to recover spatial resolution and utilizes a Unit PointNet to further extract global features for each point, ultimately completing the point cloud segmentation (Qi et al., 2017b).

#### 2.3.2 Improved PointNet++ network architecture

**Figure 6:**
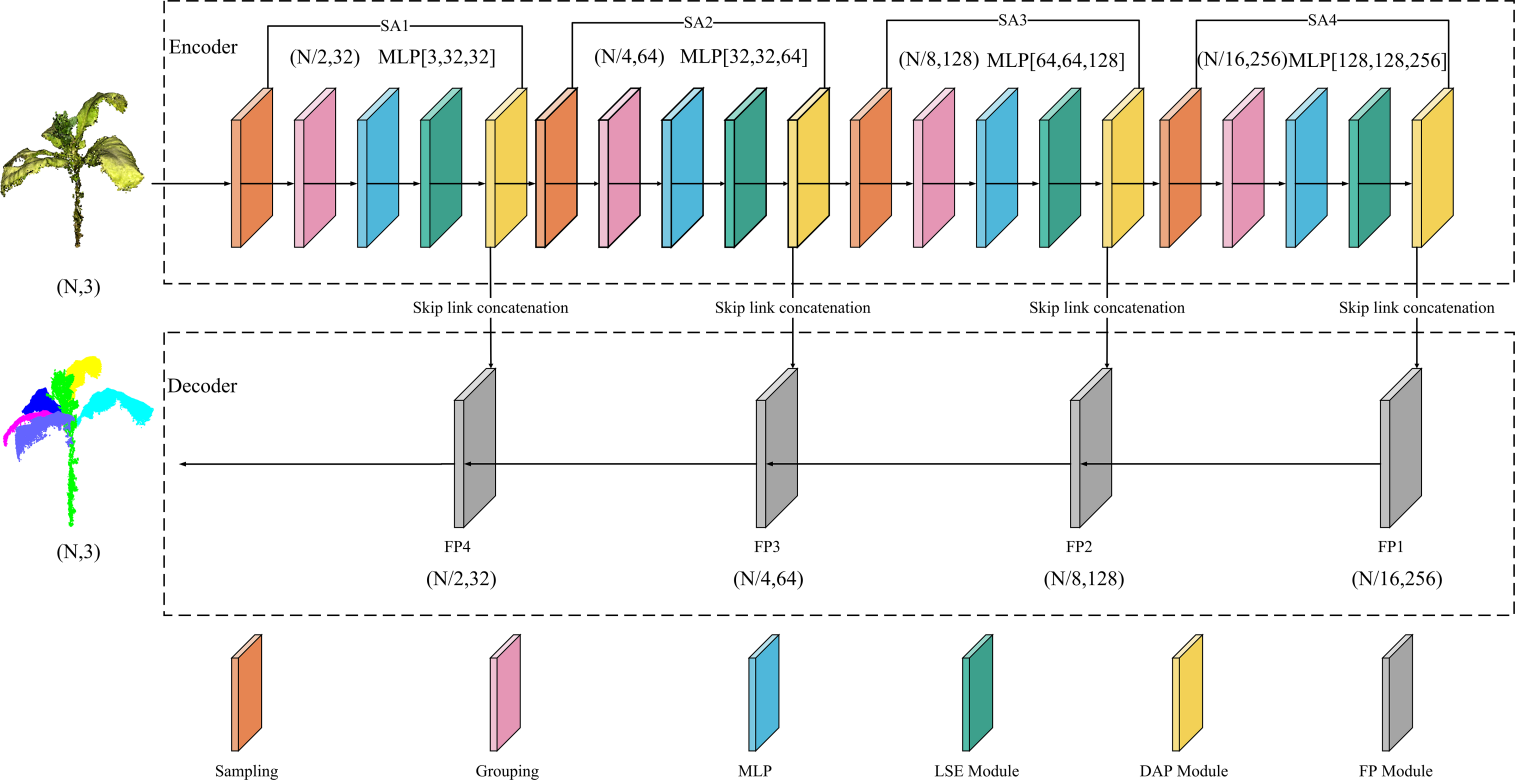
Improved PointNet++ network architecture.

To enhance the spatial alignment capability of the model, the input point cloud data were first normalized to a unified coordinate scale. The overall architecture of the improved PointNet++ consists of an encoder and a decoder (**Figure 6**).

The encoder is composed of multiple stacked SA modules. Each module incorporates a sampling layer, a grouping layer, an MLP layer, an LSE module, and a DAP module. These elements are employed to extract point cloud features layer by layer, encode spatial positional relationships, and conduct local feature aggregation.

Specifically, in the encoder process, the input is tobacco point cloud data in the (N, 3) format, where N indicates the number of points and 3 represents the feature dimension. The network utilizes the FPS strategy to determine the number of central points. To balance the point cloud information between shallow and deep layers, each layer retains half of the central-point count from the previous layer using this strategy. Subsequently, each central point selects its nearest neighboring points to construct local regions. Next, feature dimension elevation is carried out via the MLP to extract deep-level point cloud information. Finally, point position information is enhanced through the LSE and DAP modules, thereby obtaining key features of the entire tobacco plant.

The decoder adopts the original FP module to perform step-by-step interpolation recovery on the downsampled features, reconstructing a high-resolution point cloud structure. Ultimately, accurate segmentation of the stem and leaf structures in the tobacco point cloud is achieved.

#### 2.3.3 Local spatial encoding module

**Figure 7:**
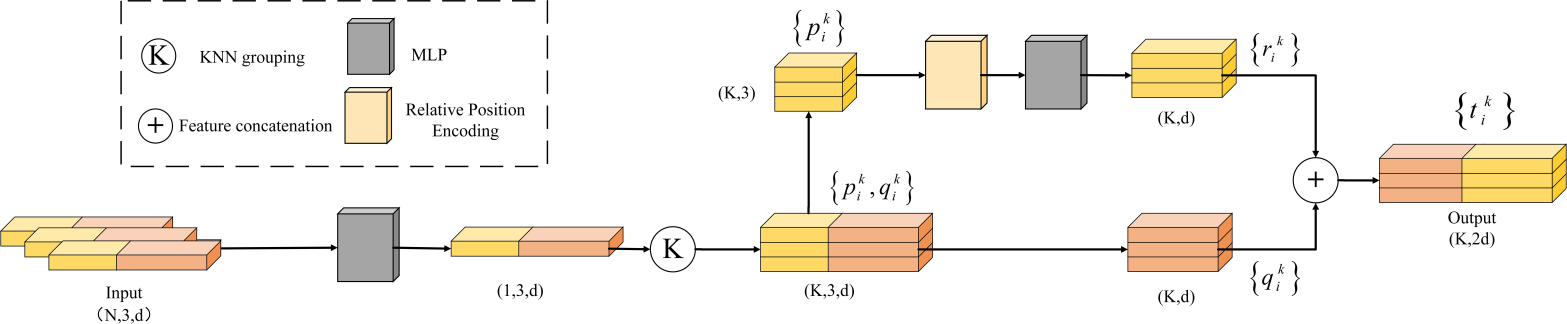
Structure of the LSE module.

This study introduces the LSE module (**Figure 7**), which explicitly encodes the local spatial relationships between center points and neighboring points to enhance point cloud positional information (Hu et al., 2020; Cheng et al., 2021).

The specific implementation steps of the module are as follows:

First, the input point cloud is represented in the format of (N, 3, d), where N is the number of points, each containing 3D coordinates (x,y,z) and d-dimensional features.

Next, after constructing local regions using KNN grouping, the Euclidean distances between each center point and its K nearest neighbors are calculated. The absolute coordinates of the center and neighboring points, their relative coordinates, and the corresponding distances are concatenated to form the relative position encoding. This encoding is then passed through an MLP for nonlinear mapping, with the output dimension adjusted to match the original feature size (Equation 3).

Finally, the encoded features are concatenated with the original features to form the position-enhanced feature vector (Equation 4). This encoding method, by fusing positional information with original features, provides an enhanced representation for each neighboring point, thereby clearly expressing the local structure of the center point and improving the network’s spatial perception capabilities.


(3) $$r_{i}^{k}=MLP\left(\left(p_{i}⊕p_{i}^{k}\right)⊕\left(p_{i}-p_{i}^{k}\right)⊕d\left(p_{i},p_{i}^{k}\right)\right)$$

(4) $$t_{i}^{k}=r_{i}^{k}⊕q_{i}^{k}$$

where $r_{i}^{k}$ ​ represents the encoded feature; $p_{i}$ represents the feature of the center point;​ $p_{i}^{k}$ represents the feature of the neighboring point; $t_{i}^{k}$ ​ represents the position-enhanced feature; $q_{i}^{k}$ ​ represents the original feature; ⊕ represents feature concatenation; $\left(p_{i}⊕p_{i}^{k}\right)$ ​ represents the concatenation of the absolute coordinates of the center and neighboring points; $\left(p_{i}-p_{i}^{k}\right)$ ​ represents their relative coordinates; and $d\left(p_{i},p_{i}^{k}\right)$ represents the Euclidean distance between them.

#### 2.3.4 Density-aware pooling module

**Figure 8:**
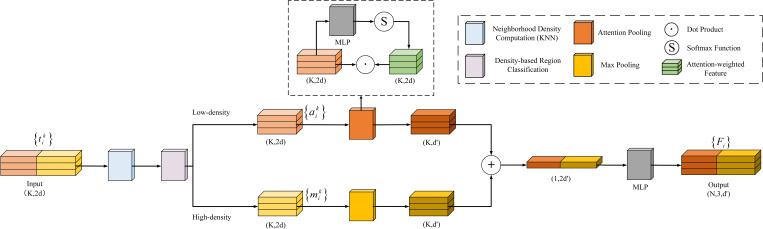
Structure of the DAP module.

After enhancing the local spatial information of the point cloud with the LSE module, this study introduces a Density-Aware Dynamic Pooling (DAP) module (**Figure 8**) to further extract key feature information. Traditional max pooling effectively extracts prominent features but ignores the relationships between features. Attention pooling can adaptively focus on key features but suffers from redundancy in high-density regions.

To combine the advantages of both, this study uses KNN to compute the neighborhood density of the center point after position enhancement (Equation 5), and normalizes the density values of all points (Equation 6), mapping them to a unified range. Then, a relative density-based strategy is introduced: if the normalized density of the center point is greater than the average density of the points in the region, it is classified as a high-density region (Equation 7); otherwise, it is classified as a low-density region (Equation 8). This strategy dynamically selects the pooling method based on the density values, adaptively processing features from different density regions, thereby improving the model’s robustness to density variations.


(5) $$D_{i}=\frac{1}{K}\sum_{k=1}^{K}\frac{1}{d\left(p_{i},p_{i}^{k}\right)+\in }$$

(6) $${\tilde{D}}_{i}=\frac{D_{i}-D_{min}}{D_{max}-D_{min}}$$

(7) $$i\in High\;:{\tilde{D}}_{i}>\frac{1}{K}\sum_{k=1}^{K}{\tilde{D}}_{i}^{k}$$

(8) $$i\in Low\;:{\tilde{D}}_{i}\leq \frac{1}{K}\sum_{k=1}^{K}{\tilde{D}}_{i}^{k}$$

where $D_{i}\;$ represents the neighborhood density of the center point; K represents the number of neighboring points; ∈ represents a small constant added to prevent division by zero; ${\tilde{D}}_{i}$ ​ represents the normalized density of the point; $D_{min}$ and $D_{max}\;$ represent the minimum and maximum densities in the entire point cloud, respectively; $\frac{1}{K}\sum_{k=1}^{K}{\tilde{D}}_{i}^{k}\;$ represents the average normalized density of the neighboring points.

Different pooling strategies are employed for local feature extraction in regions with varying densities:

Low-density regions: An attention pooling strategy is applied. First, the features of neighboring points are fed into a shared function composed of an MLP and softmax to compute attention scores. These scores are then used to weight the local features through element-wise multiplication (dot product), and the weighted features are summed to obtain the aggregated local representation (Equation 9). This strategy adaptively emphasizes key features in sparse areas and improves the feature representation in low-density regions.

High-density regions: A max pooling strategy is adopted. In dense regions such as stems or leaves, max pooling effectively suppresses redundant information and extracts the most prominent structural features within the local area (Equation 10).

Finally, the features extracted from both regions are concatenated along the channel dimension and passed through an MLP for feature fusion and dimensionality reduction, yielding the enhanced point cloud representation (Equation 11). This module fully leverages the structural characteristics of regions with different densities and achieves fine-grained local feature extraction.


(9) $$F_{att}=\sum_{k=1}^{K}Softmax\left(g\left(a_{i}^{k};W\right)\right)\cdot a_{i}^{k}$$

(10) $$F_{max}=max_{k=1}^{K}\left(m_{i}^{k}\right)$$

(11) $$F_{i}=MLP\left(F_{att}⊕F_{max}\right)$$

where $D_{i}\;$ represents the neighborhood density of the center point; K represents the number of neighboring points; ∈ represents a small constant added to prevent division by zero; ${\tilde{D}}_{i}$ represents the normalized density of the point; $D_{min}$ and $D_{max}\;$ represent the minimum and maximum densities in the entire point cloud, respectively; $\frac{1}{K}\sum_{k=1}^{K}{\tilde{D}}_{i}^{k}\;$ represents the average normalized density of the neighboring points.

### 2.4 Tobacco phenotypic parameter calculation

#### 2.4.1 Coordinate de-normalization

To ensure consistent data scaling, coordinate de-normalization was applied to the normalized point cloud data during the computation process, restoring it to the original scale to ensure the accuracy of the results. The core of de-normalization involves using the minimum value and range recorded during normalization to fix the point cloud coordinates to the actual physical space. Specifically, all normalized point cloud coordinates (x, y, z) are de-normalized to align with the measured data (Equation 12).


(12) $$x_{orig}=x_{norm}\times R_{x}+min_{x}$$

where $x_{orig}$ represents the original coordinate value; $x_{norm}$ represents the normalized coordinate value within the range [0,1]; $R_{x}$ represents the value range for the corresponding dimension; and $min_{x}$ represents the minimum value of that dimension.

#### 2.4.2 Phenotypic feature extraction

**Figure 9:**
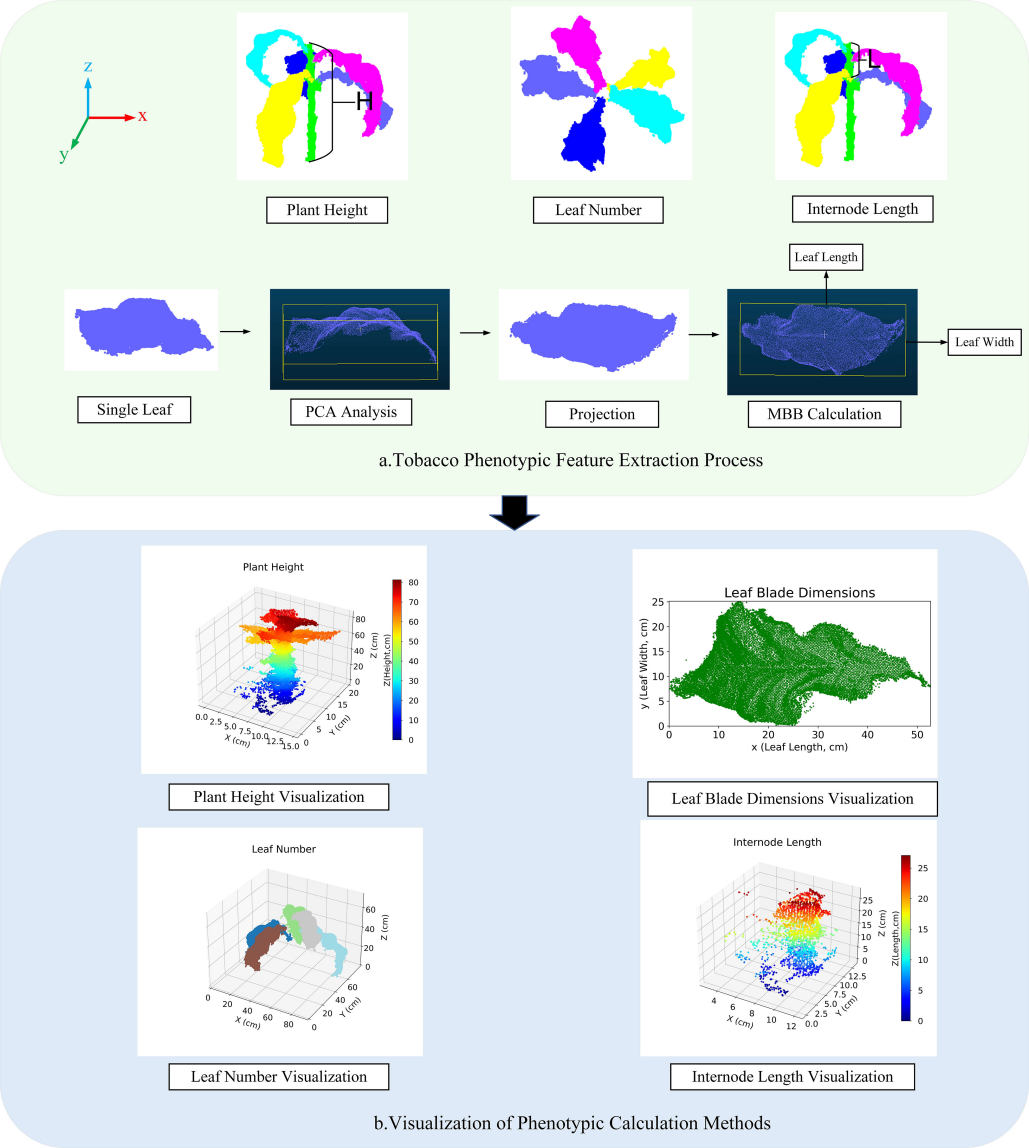
Visualization of phenotypic feature calculation and methods. **(a)** Tobacco Phenotypic Feature Extraction Process; **(b)** Visualization of Phenotypic Calculation Methods.

Extraction process of tobacco phenotypic features (Patel et al., 2023) (**Figure 9**).

Plant Height: The height difference between the highest and lowest points in the segmentation results is used as the predicted value for plant height (Equation 13).


(13) $$H_{pred}=Z_{max}-Z_{min}$$

where $H_{pred}$ represents the predicted plant height; $Z_{max}\;$ and $Z_{min}$ represent the z-coordinates of the highest and lowest points, respectively.

1. Leaf number: Based on the label grouping of the segmentation results, the point cloud data is processed for region identification, with each leaf treated as an independent region and numbered. Different colors correspond to different labels.
2. Leaf length and leaf width: These are calculated using Principal Component Analysis (PCA) combined with the Minimum Bounding Box (MBB) method. First, based on the segmentation results, PCA is applied to compute the main direction of the leaf point cloud, and the point cloud is projected onto the XY plane. Then, the minimum bounding rectangle on this plane is calculated, with the longest and shortest sides defined as the approximate values for leaf length and leaf width, respectively.
3. Internode length: The vertical distance between the labels of the upper and middle leaves. The predicted value is calculated from the vertical distance between the corresponding leaf label regions in the segmentation results.

#### 2.4.3 Visualization of phenotypic calculation methods

This section employs visualization techniques to present the phenotypic features of tobacco plants **Figure 9**.

Figure A visually illustrates the distribution of plant height using a color gradient; Figure B shows the relationship between leaf length and width; Figure C distinguishes the leaf number of different plants using color differences; and Figure d reveals the internode length of the plants, displaying the vertical arrangement density of the leaves.

### 2.5 Network training and evaluation metrics

#### 2.5.1 Experimental environment and parameter settings

To achieve accurate point cloud segmentation of tobacco plants, this study reconstructed the experimental framework from two perspectives: experimental environment configuration and model training strategy design. **Table 2** presents the hardware and software configurations used in the experiment. **Table 3** presents the key hyperparameters optimized for the segmentation experiments.

**Table 2:** Experimental environment parameters.

Parameters	Type
CPU	Intel(R) Core(TM) i5-13600KF
GPU	NVIDIA RTX4070 Ti SUPER
CUDA	CUDA 11.8
Operating System	Windows 11
Programming Language	Python 3.8
Development Environment	Pycharm2023
Deep Learning Framework	Pytorch

**Table 3:** Hyperparameters for the segmentation experiments.

Parameters	Value
Max_epoch	50
Batch_size	4
Num_points	8192
k	16
Learning_rate	0.001
Decay_rate	1e-4
Lr_decay	0.7

#### 2.5.2 Segmentation evaluation metrics

In this study, the segmentation results of the tobacco point cloud dataset were quantitatively evaluated using four performance metrics: Overall Accuracy (OA), Intersection over Union (IoU), and mean Intersection over Union (mIoU).

OA assesses the overall classification performance of the model by calculating the ratio of correctly classified points to the total number of points (Equation 14).

IoU is used to measure the segmentation performance for each class, defined as the ratio of the intersection to the union between the predicted region and the ground-truth region of a specific class (Equation 15).

The mIoU reflects the overall segmentation performance across all classes and is calculated as the average IoU over all categories (Equation 16).


(14) $$OA=\frac{\sum_{i=0}^{k}P_{ii}}{\sum_{i=0}^{k}\sum_{j=0}^{k}P_{ij}}$$

(15) $$IoU=\frac{P_{ii}}{\sum_{i=0}^{k}P_{ij}+\sum_{j=0}^{k}P_{ji}-P_{ii}}$$

(16) $$mIoU=\frac{1}{k+1}\sum_{i=0}^{k}\frac{P_{ii}}{\sum_{i=0}^{k}P_{ij}+\sum_{j=0}^{k}P_{ji}-P_{ii}}$$

where k represents the total number of classes; $P_{ii}$ represents the number of points correctly predicted as belonging to class i  ; $P_{ij}$ represents the number of points from class  i incorrectly predicted as belonging to class j ; $\sum_{i=0}^{k}P_{ij}$ represents the total number of points in class i (including both correctly and incorrectly classified points).

#### 2.5.3 Phenotypic evaluation metrics

This study adopts the coefficient of determination (R²) and the root mean square error (RMSE) to evaluate model performance.

R² assesses the degree of fit between the predicted and measured values (Equation 17), where a value closer to 1 indicates a better fit.

RMSE quantifies the average deviation between the predicted and measured values and reflects the magnitude of prediction error, with units consistent with the original data (Equation 18).


(17) $$R^{2}=1-\frac{\sum_{i=1}^{n}{\left(y_{i}-{\hat{y}}_{i}\right)}^{2}}{\sum_{i=1}^{n}{\left(y_{i}-\bar{y}\right)}^{2}}$$

(18) $$RMSE=\sqrt{\frac{1}{n}\sum_{i=1}^{n}{\left(y_{i}-{\hat{y}}_{i}\right)}^{2}}$$

where $y_{i}\;$ represents the measured value, ${\hat{y}}_{i}$ represents the predicted value, $\bar{y}\;$ represents the mean of the measured values, and *n* represents the total number of data samples.

## 3 Results

### 3.1 Accuracy analysis of the improved PointNet++ tobacco stem-leaf segmentation model

To validate the effectiveness of the proposed method, under consistent experimental conditions, the segmentation accuracy of the improved PointNet++ model was compared not only with that of existing mainstream point cloud segmentation models but also with that of the classic DBSCAN clustering method for tobacco stem - leaf segmentation, as shown in **Table 4**.

**Table 4:** Comparison of results for different models on the tobacco dataset (%).

Model	Stem IoU	Leaf IoU	mIoU	OA
DBSCAN	80.31	56.26	68.29	72.75
DGCNN	72.82	62.13	67.48	78.41
PointNet	71.29	64.30	67.80	79.52
RandLA-Net	74.64	67.43	71.04	81.56
GrowSP	84.40	74.40	79.40	85.16
PointNeXt	85.12	79.37	82.25	88.30
PointNet++	87.70	89.14	88.42	90.13
Improved PointNet++	**92.60**	**95.33**	**93.97**	**95.25**

Bold values indicate the best performance across all experimental conditions.

The experimental results show that the improved PointNet++ model performs best in segmentation accuracy, with mIoU and OA reaching 93.97%, 95.25%, respectively. Compared to PointNet++ (Qi et al., 2017b), these two metrics improved by 5.55%, 5.12%, respectively, demonstrating its advantages in fine-grained feature extraction and structural recognition.

In terms of class segmentation performance, the Improved PointNet++ model achieved Stem IoU of 92.60% and Leaf IoU of 95.33%, respectively, significantly outperforming other comparison models. Specifically, the DBSCAN (Gaonkar and Sawant, 2013) clustering exhibits certain performance in stem segmentation but performs poorly in terms of Leaf IoU and OA metrics. The DGCNN (Phan et al., 2018) and PointNet (Qi et al., 2017a) models are limited in local feature extraction, resulting in relatively poor overall segmentation performance; RandLA-Net (Hu et al., 2020) exhibits some instability in leaf segmentation; although GrowSP (Zhang et al., 2023) outperforms the first three models (in comparison) in terms of stem and leaf IoU, there is still significant room for improvement. The results of PointNeXt (Qian et al., 2022) and PointNet++ are relatively close, but the Leaf IoU of PointNet++ is higher than that of PointNext.

Therefore, the incorporation of the LSE and DAP modules has enhanced the accuracy and robustness of the model in tobacco stem-leaf segmentation, particularly in category differentiation and feature aggregation.

Meanwhile, **Table 5** presents the results of ablation experiments conducted to further assess the contribution of the LSE and DAP modules to the overall performance of the improved model. Compared to the baseline model, the inclusion of the LSE module led to increases of 3.88% and 3.34% in mIoU and OA, respectively, indicating that local spatial representation was effectively enhanced. This improvement alleviates the issue of incomplete spatial structure expression caused by sparsely distributed point clouds.

**Table 5:** Comparison of results for different modules on the tobacco dataset (%).

Model	Stem IoU	Leaf IoU	mIoU	OA
PointNet++ (Baseline)	87.70	89.14	88.42	90.16
+LSE	92.05	92.56	92.30	93.50
+DAP	90.72	92.76	91.74	92.20
Improved PointNet++	**92.60**	**95.33**	**93.97**	**95.25**

Bold values indicate the best performance across all experimental conditions.

Subsequently, the incorporation of the DAP module resulted in additional improvements of 3.32% and 2.04% across the two evaluation metrics, respectively. These results suggest that density-aware pooling effectively optimizes local feature extraction across regions with varying point densities, addressing the limited adaptability of conventional pooling strategies in both high- and low-density areas.

Ultimately, the improved PointNet++ model, incorporating both LSE and DAP modules, achieved the best overall performance, with a mIoU of 93.97% and OA of 95.25%. In particular, the IoU values for stem and leaf segmentation improved by 4.90% and 6.19%, respectively, confirming that the synergy between the LSE and DAP modules significantly strengthens the model’s ability to extract structural features and improves the accuracy of complex plant structure recognition.

### 3.2 Visualization of tobacco stem-leaf segmentation results

**Figure 10:**
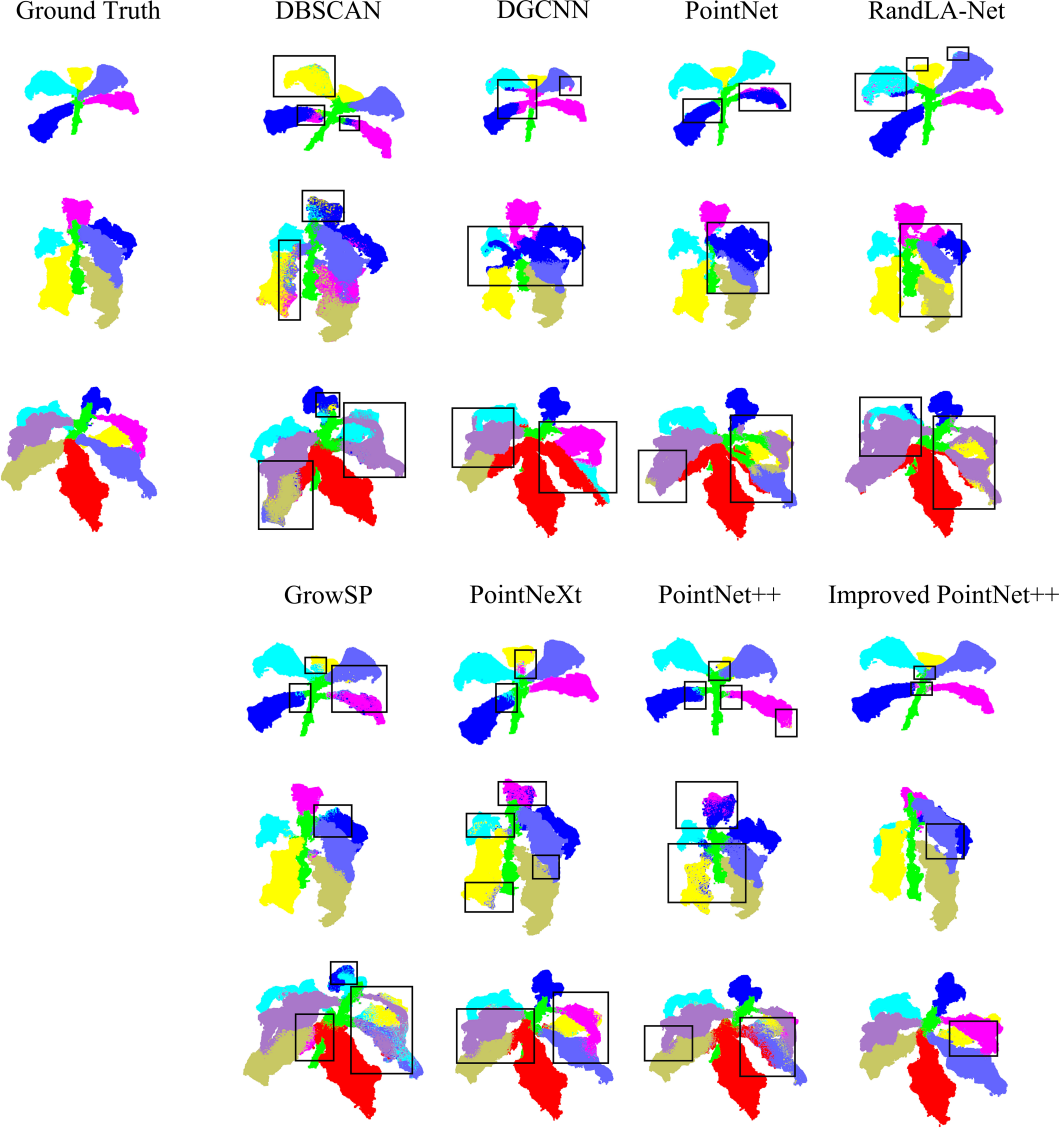
Visualization comparison of segmentation results.

To visually demonstrate the phenotypic characteristics of tobacco in the point cloud segmentation task, three tobacco plants with varying levels of structural complexity were selected. The visualized results of five segmentation models are compared (**Figure 10**), clearly reflecting the differences between the ground truth and the predicted results.

For plants with simple structures, the DBSCAN algorithm exhibits leaf segmentation errors. The prediction results of the other models are relatively close to the ground truth labels, but there are still a small number of mis-segmentations in the details.

As the complexity of plant structures increases, the DBSCAN algorithm shows limitations in leaf segmentation, with relatively severe mis-segmentation situations. the segmentation results displayed by the DGCNN and PointNet models are relatively coarse, with some regions not matching the ground truth, and they also have limitations in capturing different structural boundaries. RandLA-Net exhibits some inconsistencies, especially in the leaf regions, where there are mismatches between the color-coded segments and the ground truth. The GrowSP model shows improvements compared to the first three models, but there are still deviations between the segmentation results in some regions and the ground truth, indicating room for improvement. PointNeXt demonstrates more accurate segmentation, approaching the ground truth in many regions. PointNet++ further enhances the segmentation accuracy, with better alignment between the segmented parts and the ground truth.

In contrast, the improved PointNet++ achieves better segmentation performance, outperforming other models in small structure regions. It demonstrates superior segmentation ability in dense leaf regions, highlighting its advantages in point cloud segmentation tasks for complex structures.

### 3.3 Extraction results of tobacco phenotypic parameters based on the improved model

**Figure 11:**
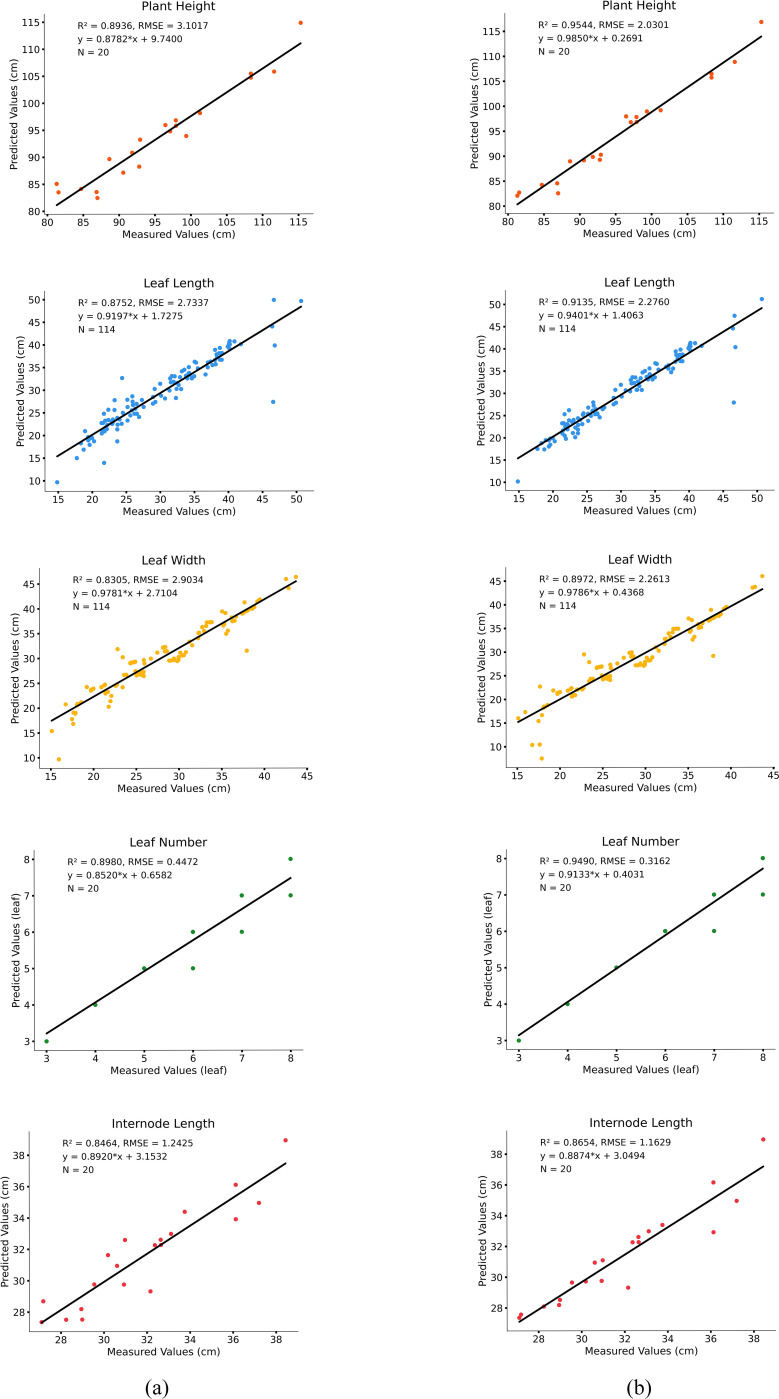
Comparison of predicted values and measured values. **(a)** computed results from the PointNet++ model; **(b)** computed results from the improved PointNet++ model.

This study predicts five key phenotypic parameters of tobacco (**Figure 11**).

The results show that the model performs well in predicting plant height, leaf length, leaf width, and leaf number, with high accuracy. Specifically, the R² value for the measured and predicted plant height is 0.95, with an RMSE of 2.03 cm, indicating a small deviation between predicted and measured values and accurately reflecting the trend in plant height variation. The prediction accuracies for leaf length and leaf width are 0.91 and 0.89, with RMSE values of 2.27 cm and 2.26 cm, respectively, with leaf length showing slightly better fitting performance than leaf width. Overall, the model exhibits low prediction errors for leaf morphological features and can consistently estimate leaf growth parameters. For leaf number estimation, the model achieves a fitting accuracy of 0.94 and an RMSE of 0.31 cm, while the internode length prediction has an R² of 0.86 and an RMSE of 1.16 cm.

As demonstrated in **Table 6**, the improved PointNet++ model exhibits superior fitting performance compared to the PointNet++ model across all phenotypic prediction tasks. The R^2^ analysis reveals consistent improvements in the five tobacco phenotypic traits, with enhancements of 0.06, 0.04, 0.06, 0.05, and 0.02, respectively. The modified model also demonstrates significant advantages in prediction accuracy, showing error reductions of 1.07 cm in plant height, 0.46 cm in leaf length, 0.64 cm in leaf width, 0.13 in leaf number, and 0.08 cm in internode length. These results substantiate the improved model’s enhanced capability in handling complex overlapping leaf scenarios. The substantial reduction in prediction errors across all phenotypic parameters validates the effectiveness of both the LSA module for feature extraction and the DAP module for structural recognition.

**Table 6:** Calculated results for tobacco phenotypic indicators.

Indicator	Method	Plant height	Leaf length	Leaf width	Leaf number	Internode length
R2	PointNet++	0.89	0.87	0.83	0.89	0.84
	Improved PointNet++	**0.95**	**0.91**	**0.89**	**0.94**	**0.86**
RMSE (cm)	PointNet++	3.10	2.73	2.90	0.44	1.24
	Improved PointNet++	**2.03**	**2.27**	**2.26**	**0.31**	**1.16**

Bold values indicate the best performance across all experimental conditions.

## 4 Discussion

### 4.1 3D reconstruction accuracy improvement with multi-view imaging and point cloud preprocessing

Due to self-shadowing effects, leaf overlap, and a lack of depth information, traditional 2D imaging techniques based on UAV remote sensing struggle to effectively extract low-noise and evenly distributed plant point cloud features from 2D images. Multi-view imaging was employed in the UAV flight mission to address this issue. As shown in **Figure 3**, images were captured from multiple viewpoints and densely reconstructed, effectively capturing the complex morphological details of tobacco plants (Hu et al., 2020). Furthermore, ground points and outliers in the reconstructed tobacco point cloud interfere with canopy segmentation and subsequent phenotypic extraction, leading to significant errors in phenotypic extraction. To tackle the issue of ground point interference, the RANSAC algorithm was employed to remove ground points, as illustrated in **Figure 3**. Through multiple iterations, the RANSAC algorithm randomly selects point sets to fit a plane model and determines ground and non-ground points based on their distance from the plane, enabling ground point identification and segmentation (Ghahremani et al., 2021; Harintaka and Wijaya, 2023). This effectively mitigates ground interference and noise in field point clouds, improving the completeness of individual tobacco plant point clouds.

**Figure 4** illustrates the overall workflow of individual tobacco plant point cloud preprocessing. The statistical filtering method reduces redundant information while preserving the overall structural features. On this basis, a uniform downsampling method is applied to reduce point cloud density and improve subsequent processing efficiency. Additionally, the preprocessed point cloud is manually annotated to provide precise semantic labels for the supervised learning model, laying the foundation for the subsequent 3D segmentation task. The results indicate that this method effectively resolves ground interference and noise issues in field point clouds, enhancing the completeness of individual tobacco plant point clouds.

### 4.2 Application of the improved PointNet++ in tobacco stem-leaf segmentation

Traditional point cloud processing methods, such as clustering analysis and skeleton extraction, often rely on manually set thresholds and multi-stage processing pipelines for tobacco stem-leaf segmentation. However, these methods struggle to achieve stable segmentation, particularly in the presence of complex plant structures and overlapping leaves (Miao et al., 2021). In contrast, deep learning models offer end-to-end feature learning capabilities, enabling automatic extraction and differentiation of stem and leaf shape and texture features. This reduces reliance on manual expertise and enhances the model’s generalization ability in complex environments.

In this study, the LSE and DAP modules were introduced to the original PointNet++ model. The LSE module enhances the model’s ability to perceive local positional information, while the DAP module allows the model to extract stable and representative local features even in sparse or overlapping regions. The comparison experiment results in **Table 4** show that OA and mIoU improved by 5.12% and 5.55%, respectively, compared to the original PointNet++. This improvement demonstrates that the introduced modules effectively compensate for PointNet++’s shortcomings in fine-grained stem-leaf feature extraction, enhancing the model’s segmentation ability and robustness in complex agricultural point cloud structures.

To further validate the individual contributions and synergistic effects of the introduced modules, an ablation experiment was conducted. The results in **Table 5** show that the model’s OA and mIoU improved compared to the original architecture, regardless of whether the LSE or DAP module was introduced alone, confirming the effectiveness of each module in its respective function.

### 4.3 Phenotypic parameter analysis

Building on the implementation of stem-leaf segmentation, this study further calculated the phenotypic parameters of tobacco plants. As shown in **Table 6**, the study achieved the automated extraction of key phenotypic traits, including plant height, leaf length, leaf width, leaf number, and internode length. The phenotypic traits obtained by this method show a strong correlation with manual measurements (R² > 0.86). Compared to traditional manual phenotyping, this method demonstrates improvements in both efficiency and stability. Manual measurements typically rely on manual recording, which is time-consuming, inefficient, and prone to human error, making it difficult to meet the high-throughput demands of large-scale field phenotyping research (Li et al., 2022). In contrast, this study leverages a 3D point cloud model combined with semantic segmentation results to automate the extraction of phenotypic parameters, significantly enhancing data acquisition speed.

In addition, the method considers spatial scale consistency during parameter extraction by performing coordinate de-normalization on the segmentation results, ensuring the comparability of the calculated phenotypic parameters in real-world dimensions. Compared to the common point selection errors and reading fluctuations in manual measurements, this method performs automatic identification and measurement based on structural logic, enhancing the stability of parameter computation.

### 4.4 Future work

The model still faces some limitations when dealing with complex plant structures. When processing tobacco plants at different growth stages or with intricate morphologies, the segmentation accuracy of the model may be affected, particularly in predicting fine structures such as overlapping leaf regions and internode length, where some errors still exist.

Future research will focus on optimizing local feature extraction and phenotypic parameter calculation, particularly improving the segmentation accuracy of complex tobacco stem-leaf structures and the precision of leaf length, leaf width, and internode length measurements. This will further enhance the model’s generalization ability across different growth stages and tobacco varieties. Additionally, the tobacco point cloud dataset will be expanded to include various growth environments and stages, thereby strengthening the model’s robustness and adaptability while optimizing data annotation strategies. Furthermore, to validate the model’s generalizability, future studies will explore its application to other economic crops, assessing its suitability for point cloud segmentation and phenotypic calculation tasks in different crops, thereby enhancing its potential for agricultural intelligence and precision phenotypic analysis.

## 5 Conclusion

This study presents a tobacco three-dimensional phenotypic analysis method tailored for field applications, covering key aspects such as data collection, 3D reconstruction, stem-leaf segmentation, and phenotypic parameter extraction. By integrating multi-view imaging with SFM-MVS technology, tobacco plant models were successfully reconstructed, providing a reliable approach for non-contact data acquisition in field environments.

Building upon this, an improved PointNet++ segmentation model was proposed, which enhanced the accuracy of tobacco stem-leaf recognition. Based on the segmentation results, the automated extraction of phenotypic parameters, including plant height, leaf length, leaf width, leaf number, and internode length, was achieved. The predicted values showed good correlation with the measured values (R² > 0.86), demonstrating the method’s potential for practical applications.

Additionally, this method is non-destructive and highly adaptable, making it suitable for phenotypic monitoring and variety evaluation in large-scale planting areas, thus providing technical support for the application of intelligent tobacco phenotyping in agricultural production.

## Data Availability

The raw data supporting the conclusions of this article will be made available by the authors, without undue reservation.
